# Selections

**Published:** 1816-07

**Authors:** 


### PART III.

### SELECTIONS.

### FOREIGN WORKS ON MEDICAL JURISPRUDENCE.

*Medico-legal Examination of Bodies.*—In our last Number, we stated the general principles which should regulate the conduct of the practitioner in the medico-legal inspection of bodies. A more detailed account of the operation itself, a methodic exposition of its different stages, and of those points to which, during its performance, the attention of the inquirer should be particularly directed, may not prove destitute of interest or utility. Thus too may we display the high consideration which medical jurisprudence has acquired upon the continent—the labour and minutenes with which every branch of it is there cultivated; and, perhaps, arouse, by the force of example, the dormant energy and emulation of our countrymen. The present article, therefore, although derived from other sources,* may be regarded as a continuation of our last on the same subject; and will, with it, form a suitable prelude to the general and extended view which we are about to take of forensic medicine, in this department of our Journal.

The business of anatomical inspection may be conveniently divided into three stages,—Preliminary measures; External examination; and the act of Dissection itself. We shall proceed to consider them in succession; avoiding, as much as possible, all prolixity and needless repetition.

1. *Preliminary measures.*—Whenever it may be necessary to defer the dissection of a body; whenever the operation cannot be terminated at one sitting, or the viscera are detached for separate examination, the ravages of the putrefactive process, which would otherwise render the research dubious or impracticable, should be carefully guarded against. With this view, the body, if it may be removed, should be deposited in a cool place, or covered with ice: it may be sprinkled with spirits or aromatic powders; or, if long preservation be an object, soaked with a strong solution of sea-salt and alum, or enveloped in charcoal powder, or dry sandy earth, capable of absorbing the moisture of the putrid fermentation. All

* The works from which the materials of the present article have been drawn, are principally the following:

*Manuel d'Autopsie cadaverique Medico-legale*, par M. Marc, (from the German of Roose).

*Medecine Legale, &c.* de P. A. O. Mahon.

*Traité de Médecine Legale, &c.* par F. E. Foderé; and

*Traité des Poisons, &c.* par M. P. Orfila,—Editors.

the orifices of the body should, at the same time, be closed, to prevent the access of atmospheric air. The viscera, separated for more minute inspection, should be immersed in the antiseptic gases. Such portions of the intestinal canal as it is intended to submit to chemical analysis, in cases of death by poison, will be best preserved in alcohol: and in the room or other place containing the body, fumes of oxy-muriatic gas should be constantly disengaged.

If the weather be warm and moist, and inflammation, gangrene, or other circumstances favouring putridity, exist, it will be impossible to check its progress. Examination, therefore, should be performed with all possible promptitude, and a current of air be made to blow over the body during dissection, whereby the noxious emanations arising from it will immediately be swept away. Fumigations with aromatic substances, tobacco, or what is yet preferable, the vapour of oxy-muriatic acid, should, at the same time, be had recourse to. These precautions are particularly requisite during the examination of a body, in which, from having long lain undiscovered or interred, putrefaction is considerably advanced.

In cases of death from external injury, it is of great importance to inspect the body in the position in which it lay when such injury was inflicted, or at the moment of death. Hence, the body should always, if possible, be viewed upon the spot where it has been discovered; lest, by removal, the attitude of the limbs or expression of the countenance should be disturbed; the condition of any wound, fracture, or lesion of the vascular system altered, or any instrument displaced, which may have caused death in an extraordinary manner. But, if removal be absolutely necessary, it should be effected with all the precautions specified in our last article, to guard against the aggravation of any existing injury; and, when the body has reached its destination, it should be placed, as nearly as possible, in the situation, wherein it lay on the reception of the wound; for we well know, that the relative situation of the internal parts is greatly influenced by the general position of the body.

It is scarcely requisite to add, that the body, at the time of actual examination, should be laid in a supine posture upon a table of convenient height, or planks properly supported; and that the clothes should be removed with all possible attention to the rules of decency. In many cases, however, the body must be completely stripped. If any wounds exist, and compresses, bandages, plasters, tents, or other surgical applications, be found upon them, the latter must be cautiously removed, and every particular respecting them be correctly specified.

2. *External examination.*—The points requiring particular notice during this stage of the inspection are, the colour of the skin; temperature of the body; rigidity or flexibility of the limbs; condition of the eyes and jaws; swelling or congestion, general or local; state of the sphincters; spots, bruises, ecchymoses, wounds, ulcers, fractures, dislocations, herniæ, prolapsus; discharges of blood or other fluids from the mouth, ears, anus, sexual organs; and, in fact, every deviation from the regular state. No brown or blue spot upon the skin should be passed over without particular notice; and, in order to determine whether such spot be a real ecchymosis, or the commencement of animal decomposition, it should be divided with the scalpel. Every part or cavity in which an injury might lie concealed from superficial view should, as we have before mentioned, be rigorously searched. An artful and expert assasin may have inflicted a deadly wound from the cavities of the mouth, nose, orbit, rectum, or sexual parts, which no common examination would detect: or vestiges of some fatal malady might be discovered there. In an infant, it is very necessary to investigate the state of the fontanelles and umbilical cord. Whenever the body cannot be recognized, all its distinguishing characters, such as stature, bulk, apparent age, colour of eyes and hair, every cicatrix, wart, and congenital mark, should be minutely ascertained and recorded.

If any external lesion exist, such as wound, contusion, fracture, or burn, we are first to specify its nature. Its situation, extent in all directions, course, and relation with the deadly instrument, if such be found, should then be accurately described. If a *wound*, we examine with what weapon it has been made; and whether or not there be contusion, inflammation, suppuration, or sphacelus; what are the vessels, nerves, muscles, viscera, divided or otherwise injured; and whether it contain any foreign body. The depth should be ascertained by probing it very delicately with lead-wire, bougie, or other flexible instrument; so that its direction and real dimensions may not be altered. If a *simple contusion*, what are the parts crushed; whether any vessel or viscus be ruptured; whether congestions or extravasation of blood or other fluid; whether the bruise indicate a force capable of producing commotion in more remote and important parts. If a *burn*, its degree and extent, the various organs which it implicates, and condition of those and the neighbouring parts. If a *fracture* or *dislocation*, whether the condition of the surrounding soft parts indicate that the injury of the bone was inflicted before or after death; whether such lesion be simple, compound, or complicated; and whether there be inflammation, suppuration, or sphacelus.

We shall terminate this article in our next Number, by a description of the third stage of medico-legal examination of bodies, or that of actual dissection.

---

### FRENCH DICTIONARY AND FOREIGN JOURNALS.

*Article, Endurcissement du Tissu Cellulaire (Induration of the the Cellular Membrane,—Induratio Telæ Cellularis).*—"This is a disease which attacks children shortly after birth; and has only attracted notice during the last forty years. Dr. Andry first wrote upon it; and, in 1787, the Royal Medical Society of Paris made it the subject of a prize. The medal, on this occasion, was adjudged to Dr. Auvity; and his Memoir is inserted among those of the Royal Society.* Various researches have since then been made on this commonly fatal malady. The infant attacked seems, as it were, frozen; and no means of warming it are successful. It generates no vital heat, and is merely penetrated, for a few moments, by warmth externally applied. The limbs display a consistence bordering on hardness, which is rarely dissipated even by the most judicious treatment. The skin is of a purple red colour. The child, motionless, scarcely cries or respires, and refuses to suck or swallow any liquid presented to it. Death takes place sooner or later, commonly in the space of from thirty hours to three days. Professor Chaussier, considering this disease not as a congelation of the fluids of the adipose structure produced by external cold, an opinion entertained by most physicians, but as a kind of solid œdema, proposes to give it the designation of *sclerema*.†

We have merely extracted from the French Dictionary this slight historical sketch, to serve as an introduction to some observations which we have lately met with in the French Journals, upon a malady of very peculiar and apparently fatal character, and hitherto little known in England. Our readers will probably recollect, that the affection is cursorily mentioned by Mr. Cross; who thinks, with undoubted correctness, that it is essentially different from tetanus.‡ We shall mingle with our facts every interesting feature which the theory or practice of continental physicians may exhibit in this singular malady. The following histories are correctly given, but in a condensed form.

*First Case.* "In March, 1801, I went with Professor Flamant to the village of Schillig, near Strasburgh, to see a child, some weeks old, belonging to an apothecary of the military hospitals. It was of a feeble constitution, and had been for some days ill. The tumefaction of the cellular membrane had the solidity of bacon, and repelled, by a peculiar elasticity, the impression of the finger. It began, as usually happens, in the lower extremities, and shortly reached the region of the pubis. Professor F. informed us, that, when arrived at this stage, the disease soon proves fatal: and, on the morrow, we received intelligence of the child's death from the father. In the preceding year, he had lost a child of the same age by this malady.

---

* *Recherches sur l'endurcissement du tissu cellulaire des enfans nouveau-nés; Mémoires, &c.* tome vii. The second and third prizes, on this occasion, were given to Drs. Hulme, of London, and *Naudeau*, St.-Etienne en-Fôret. Several other continental physicians have mentioned this malady. A short but accurate description of it, under the title of *Congelamento del grasso della cellulaire integumentale, ne' Bambini,* is given by Dr. Vacca Berlingiheri, in his *Codice elementaire di medicina pratica, sanzionato d' all' esperienza.* EDITORS.

† From the Greek adjective, σκληρος, hard.

‡ See "Medical Sketches," page 197; or our Review, page 239, of the first volume. EDITORS.

"Professor F. regards this species of coagulation as a consequence of defect of vital energy: and indeed here, independently of the external signs of constitutional weakness, the child displayed extreme torpor; had a feeble and languid pulse; and the diseased extremities were below the healthy temperature."

"Tonics, as wine, distilled aromatic waters, Hoffman's anodyne, and syrup of orange peel, were particularly recommended, as also frictions with camphorated liniment of ammonia, application of external warmth, &c. But all these means had, in this case, been employed too late; and I believe the Professor had never seen any decided benefit result from them on other occasions."

*Second Case,* was that of a weakly and diseased child, aged four months. It was first seized with symptoms of lymphatic congestion of the brain. The head became very large. There existed a marked elevation on the left parietal bone, and profound torpor. This affection was removed by mercurials and tonics. Shortly afterwards, the peculiar signs of Sclerema were developed in the lower extremities. Baths of warm decoction of sage (salvia officinalis) were employed with remarkable effect. "A very decided febrile paroxysm was excited by the first bath; and the same, but successively less distinct, was observed after the others: and, moreover, by their use the cellular induration was soon completely dissipated. Tonic medicine and diet kept up for a time the action of this remedy." The child eventually died from serous congestion of the lungs.

*Third Case.* "I was called, on the 9th of October last, to a male child, one year old, born with all the external signs of a strong constitution, and having hitherto been healthy and plump. The child had preserved its appetite and sprightliness; but, for some time past, a swelling, of peculiar character, had been perceived on the right hand and foot. It had begun, three weeks previously, on the fingers and toes, and gradually spread. On my first visit, I found the hand and foot nearly disfigured by the swelling: yet the induration had not extended much beyond the shoulder, above, or the middle of the thigh, below. The face had, for some days, been implicated in the disease, and the cheek was become large, and exhibited similar characters. On the left foot and leg also the affection was just beginning."

"The characters of this swelling were indolence and elasticity. The digestive organs appeared undisturbed; respiration free, pulse slightly and irregularly febrile. The child ate and sucked, but was tormented by constant thirst. Urine scanty; sleep longer since the face had swollen, yet without coma. No expression of remarkable suffering."

"Prescribed, that evening, immersion of the body, for fifty minutes, in strong infusion of sage; and gentle friction, meanwhile, of the swollen parts; afterwards, general frictions and a warm bed. Three grains of calomel with a little rhubarb and sugar, in honey: infusion of mint with nitre for common drink."

"10th. Morning. Restlessness after the bath, followed by copious perspiration and two stools. Swelling of the left lower extremity diminished; pulse still febrile. Another bath at nine o'clock: powder repeated. Evening. Swelling better; less stiffness; several stools. Half the powder only had been got down at mid-day. Powder and bath repeated."

"11th. Morning. Child calm; rested well nearly all night; swelling every where much diminished; bowels open. Powder and bath repeated. Evening. Swelling of the thoracic (superior) limbs very great and resistent; ascending towards their middle; always most considerable in the right. The right cheek tolerably soft; the left, hitherto unaffected; now, on the contrary, hard and swollen: inferior extremities nearly restored to their natural state. The child, somewhat more calm, constantly takes both food and medicine. Powder repeated."

12th. Morning. Night good; swelling of the superior limbs diminished both in volume and extent; that of the face nearly gone, but the left fore-arm harder and more sensible to the touch. I directed these extremities to be wrapped in flannel, moistened with warm infusion of sage; thinking that they continued more swollen, with respect to the lower limbs, from having been less exposed to the action of the bath. Bath repeated at mid-day; remedies continued; fomentations occasionally renewed. Evening. Unusual sensibility of the thoracic limbs on touch or motion, but scarcely perceptible in a state of rest. The child calm without drowsiness. No stool; countenance favourable, and little swollen. The vestiges of the affection scarcely perceptible in the abdominal (lower) extremities. In the early part of the night, great agitation and extreme thirst; the motions of the thorax, before unimpeded, became audibly oppressed; the thoracic limbs, particularly the left, evinced an exquisite sensibility; the exacerbations broken by short and imperfect intervals of tranquillity." "About four in the morning, the child appearing to slumber, its relatives, and the servant who sat up with it, fell asleep. Upon their awaking, the child was dead."

"At three o'clock, P. M. of the 13th, I examined the body. It was cold, and the tumid limbs very stiff. The swelling displayed the same elasticity as during life; it preserved, as in œdema, the impression of the finger but little, and on the back of the right hand only. The whole lumbar region, right hip, and posterior superior part of the corresponding thigh, were of a deep-brown colour; a similar spot appeared upon the back of the left hand."

"The abdomen was the only cavity which I thought necessary to open; for no signs of cerebral affection had existed; and the functions of the thorax had been unembarrassed (almost) to the last moment. The viscera were sound; the intestines of a natural colour, and without worms; the liver somewhat increased in volume; the gall-bladder full of bile."

"I made incisions in the right leg, which was first swollen; its cellular structure differed little from the ordinary state; yet there was an unusual approximation of its layers, and solidity of the fat, which appeared clotted (grumelée). From an incision of the back of the corresponding hand, a small quantity of serum oozed, resembling that of a leuco-phlegmatic swelling. The volume of these parts was entirely owing to a solid and gelatinous developement of the rete mucosum.

"The principal swelling, although most recent in its formation, was on the left arm. It possessed considerable hardness, and extended from the ends of the fingers three fourths up the arm. This part offered some resistance to the scalpel: its incision resembled that of the fat of a pig recently killed; and no serum escaped from it."

Some points of analogy, the author thinks, exist between cellular induration and croup. What those points are, we profess not to comprehend. His other observations have nothing to recommend them.*

*Fourth Case.* "A child, born November the 8th, 1813, was received on the 10th into *L'Hospice de la Maternité*, and displayed the following symptoms: Tumefaction of the feet, legs, thighs, and pubic region, with loss of motion and sensibility, redness, coldness, retraction, and decided induration: soles of the feet arched length-wise and wrinkled from right to left: cheeks swollen, cold, hard, red, yielding little to pressure; impeding, by their tumefaction, the opening of the mouth, and preventing suction. Profound stupor, resembling that of apoplexy; shrill and plaintive cry, whenever moved. Pulse imperceptible; no action in the precordial region; heat felt only at the anterior part of the thorax. The finger, applied to the anterior fontanelle, detected rather an undulatory motion than a pulsation. No stools. Two days afterwards, the disease had made a rapid progress. Superior limbs swollen. Death."

*Examination of the body.* The skin was every where covered with red and yellow spots of various extent, and irregular figure, streaked with white, which gave it the appearance of being, as it were, speckled or variegated. The cheeks, neck, and sternal region, were more red and hard. All the parts, abounding in fat, were harder, and colder than the others. Limbs bent and strongly contracted: articulations of the ankle and wrist, swollen and little stiff, allowing motion as in life."

---

* These three first cases are extracted from a paper by M. J. M. B. Bard, in a late number of the *Journal général de Médecine &c. par Sedillot.* EDITORS.

"The violet coloured parts, being cut, furnished a considerable quantity of thin, pale blood: the integuments of the posterior part of the cranium were from four to five lines thick, in some places; in others, two or three. The margins of the incision were indurated, cold, yellow, and partly composed of a granulated, hard fat, which filled the interstices of the cellular membrane. The skin was hard, and separated readily from the muscles, particularly on the legs; from whence it was removed in the form of a buskin: the thighs were in the same state. The inguinal and axillary glands were numerous, large as lentils, flat, elongated, without the induration, coldness, or red colour of the other parts. The glands, said to be found in the *fossa canina*,* were merely lumps of fat. The eye-lids were sound, but injected, as well as the labia (pudendi)."

*Cranium.* "Superior longitudinal sinus filled with thick black blood; all the meningeal vessels gorged; the membranes inflamed; the lateral ventricles containing about a thimble full of bloody serum; cerebellum somewhat soft and red; *medulla oblongata* slightly indurated; *basis cranii* filled with blood and serum; contents of the vertebral canal apparently inflamed."

*Thorax.* Pleura strongly adherent to the posterior surface of the sternum, to the pericardium, lungs, and particularly the thymus gland, which was very large. Pericardium intimately connected with the superior surface of the diaphragm, distended and enveloping the heart, of thrice the ordinary size; auricles and ventricles filled with black and coagulated blood. The lungs shrunk to the bottom of the cavity, without crepitation, and exhibiting only one point where the blood was of a vermilion colour, on the right lung: all the remainder, cut into pieces and thrown into water, sank, while that point swam. The lungs, moreover, had the appearance of liver; were of a red brown colour, heavy, and resisted pressure, without any thing of the natural crepitation. The trachea and bronchia were lined by a mucous film."

*Abdomen.* "Intestines distended by gas; mesentery gorged with reddish blood; liver voluminous, compact, of a deep red colour; gall bladder brown and distended with much blackish bile. Kidnies large, red; and wrinkled. Pancreas large and red. Stomach very small, flaccid, and traversed by numerous loaded blood-vessels."

The following are the results of "several chemical experiments which the author has made upon the skin, cellular membrane, and fat of subjects, destroyed by *sclerema*, relatively to the state of the same parts in other subjects."

"The *skin* of the diseased child was generally more red, hard, and cold; containing a greater quantity of blood in the *rete mu-*

---

* A slight depression in the surface of the superior maxillary bone, between the root of the socket of the first bicuspis, and infra orbitar foramen, and affording attachment to the *levator anguli oris* muscle. Edit.

*cosum,* and more gelatine. Exposed to air, or placed in water, it exhales a more acid smell, and becomes sooner putrid: it furnishes, on ebulition, more gelatine. The *cellular membrane* presents a greater quantity of serum, which again increases after death. This structure is difficult of inflation: it contains beneath the skin a prodigious quantity of yellowish fat, in small grains, firm, hard, and cold, with a filamentous covering: it putrefies more quickly than the healthy membrane. The *fat* is heavier than in the natural state: it crackles* beneath the fingers; and communicates to water a red tinge. The fluid evaporated affords a residue more copious by one fifth."

The author, after showing, from actual inspection, the inaccuracy of the opinion "that lungs are affected with gangrene, when they are merely *hepatized* or loaded with blood," adds: "my own observations prove that *sclerema* has only an analogy with certain indolent enlargements which occur in the extremities of persons suffering from chronic pneumonia, complicated with debility, or ossification, of the organs of circulation."

We cannot more properly close this article than with a brief description of the preventive and therapeutic measures, which the Author recommends against this destructive malady.

Protecting the body of the child from the impression of air and all cold substances, at the moment of birth, constitutes the principal prophylactic mean. The curative plan consists,

1st. In placing the child on one side, in an oblique posture, so that the head may be most raised; and laying every part in a state of flexion:

2d. Giving frequently, and in small quantities, the mother's milk, or that of another woman, recently delivered; and rendering it purgative, if necessary, by giving cathartics to the nurse. If human milk cannot be procured, that of the cow diluted;

3d. Not swathing the child; laying it with the nurse; covering it with a woolen bag which will not impede motion; and inclosing it under a kind of hoop, which may preserve it from the weight of the clothes and motions of the nurse; in frequently changing the linen, and every other attention to cleanliness;

4th. In employing to it slight friction with the hand or flannel, and afterwards wrapping it in coarse wool, dry, recently carded, just warmed, which is to be frequently changed;

5th. Keeping the child in a somewhat moist atmosphere of the temperature of from 25° to 30° Reaum: (about 56° to 67° Fah.);

6th. Applying, according to circumstances, leeches to the chest, and mustard-plasters or blisters to the part which appears first indurated;

7th. Using tepid baths of a temperature of from 28° to 34° R. (63° to 76° F.): if the debility be very great, and the disease

* The verb, in the original, is *scintille,* literally, *sparkles.* But we presume that the fat possesses, even in this disease, no such property. Edit.

stationary or indolent, administering some tonic, as old wine or a cordial potion; and

8th. Sometimes it becomes necessary to prescribe gentle laxatives, as syrup of peach-flowers, weak infusion of rhubarb, or emollient glysters."*

---

### *Dislocation of the fifth cervical Vertebra from the sixth.*†

A Carter, aged 41, of a strong constitution, and athletic, was knocked down, on the 27th of May, 1811, by a piece of timber, with which he was endeavouring to load a carriage. In this fall, the posterior part of the neck came into contact with the axle-tree. And, although the contusion had been inflicted upon this part only, he could neither rise nor move. He was immediately conveyed to *L'Hôpital Saint-Antoine*.

" Upon examination, I found the abdominal (sacral) extremities and the left thoracic (atlantal) paralysed, with considerable numbness of the right thoracic limb.

" Respiration was tight; the voice, before strong and clear, now feeble and hoarse: the intellectual faculties natural; the pupils dilated. He could, with difficulty, express himself, and respiration was very laborious. The patient complained only of slight pain in the posterior part of the neck, lumbar region, and sacrum. These regions presented no external mark of contusion: the pulse was small and concentrated. Baths, frictions, fomentations, and internal sedatives, were employed in vain; and the patient died during the night, nineteen hours after the accident.

" *Dissection.* I discovered nothing (uncommon) in the three cavities. I divided the skin and muscles of the posterior part of the neck; and found blood poured out among the muscles of this region. The effusion extended to the attachment of the *trapezius*. Upon removing this blood, I found a separation of half an inch between the fifth and sixth cervical vertebræ. The *ligamentum subflavum* was ruptured, as well as the articular ligaments. The inter-vertebral substance was much distended, and allowed the free motion of these vertebræ. The spinal marrow was covered with blackish blood, which filled all the vertebral canal. Upon examining the region of the sacrum and coccyx, where the pain had been felt, I found nothing capable of explaining its cause.

" It results from this dissection, that paralysis of the abdominal and thoracic extremities was determined by compression, and injury, of the cervical marrow."

---

* *Memoire, &c.* par M. Le Docteur Troccon.—*Bulletin de la Societé Médicale d'Emulation.* No. 11, Nov. 1815.

† Par M. Le Professeur Thillaye. *Bulletin de la Faculté de Médecine, de Paris, &c.*

### *Extraordinary Cases of Hydrocele.**

Among the numerous anatomical observations, for the most part illustrated by drawings, which we have made, and shall presently communicate to the *Faculté,* there is a certain number concerning hydrocele. Of these last, there are two, remarkable from the nature of the matter of the dropsy: they are the following.

" *First Case. Concrete Hydrocele.*—On the body of a man, aged about 60, the right side of the scrotum presented an ovoid tumour. nearly six inches long, and four in its other diameters. This opake and heavy tumour appeared, on first examination, to be a sarcocele. Yet, upon incision, we found that the flattened testis was placed at its posterior and inferior part; that the greatly-thickened *tunica vaginalis* was fibro-cartilaginous in many points, and fibrous every where else. The cavity of this membrane was filled with a white, opaque, concrete substance, resembling coagulated albumen. The tumour was formed by this substance, in quantity about one pound, and adhering to the internal surface of the membrane, which was uneven and lamellated. Slices of this substance, exposed to heat upon a plate of iron, became at first more concrete, giving out bubbles of gas with the odour of sulphuretted hydrogen; and were afterwards consumed, yielding the same smell, united to that of burnt horn."

" *Second Case. Hydrocele, containing Crystals of a peculiar fatty Substance.* We found upon the body of a man, aged about 70 years, the sacs of two inguinal herniæ. The same body presented an hydrocele of the *tunica vaginalis,* which was ovoid, and might be about the size of two fists. The sac of this hydrocele was rather thick, whitish, fibrous as it were, and formed by the tunica vaginalis and surrounding cellular structure. The testicle, perfectly sound but much flattened and extended, entered into the composition of this sac, and did not project into its interior. The sac was filled by a fluid of a clear brown colour, slightly viscous, somewhat acid in smell; in quantity about one pound. This fluid contained a considerable quantity of yellow, shining crystals, of a metallic lustre, resembling little laminæ of gold, or rather small pieces of mica held in suspension. The internal surface of the tunica vaginalis was lined with these crystals, which were incrusted in its substance, and was covered in some places by a very thin pellicle.

" The fluid, drawn from the tumor and left to itself, separated into three strata: 1st. The uppermost and most abundant might form nine-tenths of the fluid; its colour was a clear brown: 2dly. The middle was of a deepish red, resembling blood: 3dly. The lower was formed of the precipitated crystals.

---

* Par M. M. Béclard, chef des travaux anatomiques, et Jules Cloquet, prosecteur. *Bulletin de la Faculté de Médecine de Paris, &c.* 1816.

"1st. The fluid, filtered and separated from the crystals, yielded to concentrated alcohol a very thick white precipitate, which dissolved in forty times its volume of water.

"2dly. The crystals, after having been washed in several parcels of water, became perfectly white; their yellow aspect having been caused by the colour of the fluid in which they swam. They were quite transparent, generally assumed a square form, and were heavier than water. They were insoluble in cold and boiling water. They were but imperfectly soluble in cold alcohol; yet they lost their metallic lustre, and became somewhat opaque. They were very soluble in boiling alcohol, which, on cooling, deposited them in the form of numerous white flakes. They melted like wax, but at a much higher temperature; and then they acted upon paper as oil does. They burned with a white flame, leaving no residue. If brisk combustion did not take place, they gave out a white smoke, which had the smell of burning horn. They were perfectly soluble in pure potash.*

"*Conclusion.* Of the three animal substances with which this might be confounded, viz. spermaceti, the fat of dead bodies (adipocere?) and the crystalline substance of human biliary calculi, the last is that which it most strongly resembles: yet it differs from that, in possessing greater weight, and in its property of readily dissolving in the alkalies.

"Professor Thillaye has sent us a crystalline substance, which he found in a similar tumor, but with an appearance much less shining; and which exhibited, with different chemical re-agents, precisely the same phænomena as the crystalline matter of biliary calculi."

---

GOTTINGEN. At the late Anniversary of the Royal Society, Professor Blumenbach read a Lecture, purporting to be a "Specimen historiæ naturalis, ab auctoribus classicis præsertim illustratæ, eosque vicissim illustrantis;" in which he entirely confined himself to quotations from Greek and Roman Poets and Historians, excluding the ancient physicians and writers on natural history. It having already, in his early life, attracted his attention, when reading the Zoological works of Aristotle and the physiological works of Galen, that they had not quoted the works of Homer, Euripides, and others, for the sake of mere show, but from actual necessity and want of their authorities. We select the following passages as examples of the whole:

---

* We beg leave here to rectify an error, which we had remarked, but forgotten to set right ere the sheet went to press, in our Review of Orfila, page 417, lines 18-19, of our first volume. *La potasse à l'alcool* means, not "potash in alcohol," but potash perfectly pure, and prepared by solution of the alkali in alcohol, and subsequent evaporation. EDITORS.

"Ovid," says he, "describes the great irritability of the tongue, which Tereus cut out from the throat of his sister-in-law," saying,

"——— Radix micat ultima linguæ.

Ipsa jacet, terræque tremens immurmurat atræ.

Utque salire solet mutilatæ caudà colubræ,

Palpitat; ———

*Metamorphos 6.*

Dr. Blumenbach found this assertion contradicted, about a dozen years ago, in some Treatise on the Construction of the Tongue, inserted in the Philosophical Transactions. Here the interior construction of the tongue was said to be less irritable than almost any other organized part of the body. In order to clear up this point, and to put it beyond doubt, Dr. B. lost no opportunity to try the irritability of that organ, by vivisections on mammalia, and having cut out the tongue of the animal when dying, and whilst it yet remained warm, he applied to it various mechanical and chemical stimuli, and always observed very striking vibrations and convulsions to arise in the muscular fibres of its proper substance, when recently divided, yet varying in strength and duration, according to circumstances not always explainable. The heart itself, which had been cut out of an ox, just killed, and after he had died from loss of blood, lost all visible irritability whilst yet warm, seven minutes sooner than the tongue; the point of which, after the transversal cut through the anterior part, shewed such conspicuous signs of life, that a woman standing by, compared it, like Ovid, to the pieces of an eel cut asunder, *mutilatæ cauda colubræ.* To this experiment, Dr. B. adds an observation, made accidentally on a human tongue, by Dr. Chanfepié, of Hamburgh, as perfectly agreeing with it.—A boy having bitten his tongue in a fit of epilepsy in such a manner, that it was found necessary to separate the piece, Dr. C. took it in his hand and saw it move, in which motion it continued for several minutes after it had been lying in the window seat. Mechanical stimulus with a needle or with salt, had the same effect; yet, it then produced no stronger motions than it had spontaneously shewn.

Xenophon, in the fourth book of his work on the Return of the Ten Thousand, says, that many of his countryman had been blinded by the dazzling whiteness of the snow, on the snowy mountains of Armenia, and that they had endeavoured to guard themselves against it, by keeping something black (μελανϛι) before their eyes. This actuated Dr. B. to compare the accounts of travellers, as well in regard of the snowblindness itself (it being, as it were, endemical in the northern parts of both the old and new world) as also particularly concerning the various methods used for the prevention and cure of it. He likewise produced two such mechanical remedies, for the inspection of the members. One of them was a bandage for the eyes, called Kaar-yoeslik, which is generally worn by the Arabians when travelling during the winter; it is made of horse hair twisted, and so bellied as not to impede the free motion of the eye-lids. The other was a kind of spectacles, manufactured by the Esquimaux of the mission of Hofenthal at Labrador. They are carved out of that enigmatical wood used for refining metals; they are as light as a feather, the upper part projects over the lower one, for the purpose of better keeping off light and snow from above, the anterior part is also for the same purpose stained with lamp-black. They have very close oblique, yet sufficiently acute incisions, for the purpose of looking through. On the interior and lower margin, which rests upon the cheek bones, are two narrow grooves, seemingly for no other purpose than to form an aqueduct for the tears. Dr. B. having, however, frequent occasion to examine natural curiosities and preparations in a strong and clear day-light, whilst he was afflicted with a chronic and heavy affliction of the eyes, he had an opportunity of deriving great benefit from the use of these snow-spectacles, and every one that saw the author's, confirms what Ellis says, that this remedy of the Esquimaux is not alone useful against the dazzling light produced by snow, but that it may also serve as a substitute for spying-glasses. *(Communicated by Dr. Von Embden, of Hamburgh.)*

